# Supplementary material for: Enhanced Adipose Expression of Interferon Regulatory Factor (IRF)-5 Associates with the Signatures of Metabolic Inflammation in Diabetic Obese Patients
Source: Cells. 2020 Mar 16;9(3):730. doi: 10.3390/cells9030730 (PMC7140673; doi:10.3390/cells9030730)
Supplement: Supplementary file 1 [file cells-09-00730-s001.pdf]

**Table S1. Primer IDs**

| Immune marker | Primer ID |
|---|---|
| TNF-α | Hs01113624_g1 |
| IL-1β | Hs01555410_m1 |
| IL-6 | Hs00985639_m1 |
| IL-18 | Hs01038788_m1 |
| IL-23A | Hs00900828_g1 |
| CXCL8 (IL-8) | Hs00174103_m1 |
| CXCL9 | Hs00171065_m1 |
| CXCL10 (IP-10) | Hs01124251_g1 |
| CCL2 (MCP-1) | Hs00234140_m1 |
| CCL5 (RANTES) | Hs00982282_m1 |
| CCL7 (MCP-3) | Hs00171147_m1 |
| CCL11 | Hs00237013_m1 |
| CCL19 | Hs00171149_m1 |
| CCR1 | Hs00928897_s1 |
| CCR2 | Hs00704702_s1 |
| CCR5 | Hs99999149_s1 |
| TLR2 | Hs01872448_s1 |
| TLR3 | Hs01551078_m1 |
| TLR4 | Hs00152939_m1 |
| TLR7 | Hs01933259_s1 |
| TLR8 | Hs00152972_m1 |
| TLR9 | Hs00370913_s1 |
| TLR10 | Hs01935337_s1 |
| Dectin-1 | Hs01902549_s1 |
| IL-1RL1 | Hs00545033_m1 |
| FGL-2 | Hs00173847_m1 |
| CD11c | Hs00174217_m1 |
| CD68 | Hs02836816_g1 |
| CD86 | Hs01567026_m1 |
| CD163 | Hs00174705_m1 |
| CD302 | Hs00994886_m1 |
| MyD88 | Hs01573837_g1 |
| NF-κB | Hs00765730_m1 |
| IRF3 | Hs01547283_m1 |
| IRF4 | Hs01056533_m1 |
| IRF5 | Hs00158114_m1 |
| AML1 (RUNX1) | Hs00257856_s1 |
| GAPDH | Hs03929097_g1 |
